# A genetic fuzzy system for unstable angina risk assessment

**DOI:** 10.1186/1472-6947-14-12

**Published:** 2014-02-18

**Authors:** Wei Dong, Zhengxing Huang, Lei Ji, Huilong Duan

**Affiliations:** 1 Cardiology Department of Chinese PLA General Hospital, Beijing, China; 2 College of Biomedical Engineering and Instrument Science, Zhejiang University, 310008, Zhou Yiqing Building 510, Zheda road 38#, Hangzhou, Zhejiang, China; 3 IT Department of Chinese PLA General Hospital, Beijing, China

**Keywords:** Unstable angina risk assessment, Fuzzy association rule mining, Genetic algorithm

## Abstract

**Background:**

Unstable Angina (UA) is widely accepted as a critical phase of coronary heart disease with patients exhibiting widely varying risks. Early risk assessment of UA is at the center of the management program, which allows physicians to categorize patients according to the clinical characteristics and stratification of risk and different prognosis. Although many prognostic models have been widely used for UA risk assessment in clinical practice, a number of studies have highlighted possible shortcomings. One serious drawback is that existing models lack the ability to deal with the intrinsic uncertainty about the variables utilized.

**Methods:**

In order to help physicians refine knowledge for the stratification of UA risk with respect to vagueness in information, this paper develops an intelligent system combining genetic algorithm and fuzzy association rule mining. In detail, it models the input information’s vagueness through fuzzy sets, and then applies a genetic fuzzy system on the acquired fuzzy sets to extract the fuzzy rule set for the problem of UA risk assessment.

**Results:**

The proposed system is evaluated using a real data-set collected from the cardiology department of a Chinese hospital, which consists of 54 patient cases. 9 numerical patient features and 17 categorical patient features that appear in the data-set are selected in the experiments. The proposed system made the same decisions as the physician in 46 (out of a total of 54) tested cases (85.2%).

**Conclusions:**

By comparing the results that are obtained through the proposed system with those resulting from the physician’s decision, it has been found that the developed model is highly reflective of reality. The proposed system could be used for educational purposes, and with further improvements, could assist and guide young physicians in their daily work.

## Background

Unstable Angina (UA) is a kind of chest discomfort or pain that occurs in a continuous and unpredictable way [1,2]. The unstable pain can result from the disruption of an atherosclerotic plaque in narrowed coronary vessels with lessened flexibility, embolization and vasospasm. As a major type of Cardiovascular Disease (CVD), UA lays its symptoms between stable angina and acute myocardium infarction and a further sudden death [2]. While the risk of UA is high, the population of UA is huge, especially for aged people and those with associated disease such as hypertension and diabetes [3]. To this end, reliable assessment of risk levels for individual UA patients will be of significant value and interest.

A number of models for UA risk assessment have been proposed in literature. Most of these models are derived from databases of clinical trials, e.g., the Thrombolysis in Myocardial Infarction (TIMI) [4], platelet glycoprotein IIb/IIIa in unstable angina: Receptor Suppression Using Integrilin (PURSUIT) [5], and the Global Registry of Acute Coronary Events (GRACE) [6], etc. They use standard patient features that are part of the routine medical evaluation of UA patients, and lead to a score to define prognostic groups [2]. Although there are many benefits related to the design and use of these prognostic models, a number of studies have highlighted possible shortcomings [2,4]. One serious drawback is that existing models lack the ability to deal with the intrinsic uncertainty about patient features utilized in UA risk assessment. Note that vagueness is fundamental and indispensable aspects of knowledge, so as in many practical problems, the experts face vagueness in feature vectors. According to Bellman and Zadeh "much of the decision making in the real world takes place in an environment in which the goals, the constraints, and consequences of possible actions are not known precisely" [7]. Regarding UA risk assessment, many patient features are vague, and not easy to be handled by existing models. It is, therefore, necessary to develop a new UA risk assessment model to deal with vague information.

In this paper, a novel UA risk assessment model has been developed using fuzzy set theories. The proposed model represents patient features with fuzzy sets and then extracts useful information with a descriptive rule induction approach based on fuzzy systems. To derive fuzzy rules from data, the proposed model employs genetic algorithms (GAs) to learn rule base from the collected data-set. GAs are search algorithms based on natural genetics that provide robust search capabilities in complex spaces [8]. The hybridization between fuzzy systems and GAs, called genetic fuzzy system (GFS), has attracted considerable attention in the computational intelligence community [9-12]. Our main goal is to develop a novel GFS such that we derive from clinical data-set a set of assessment rules, which has good interpretability before determining an efficient assessment model in order to get high accuracy of UA risk stratification. Since the accuracy of an assessment model can be largely affected by processing vague patient features, this paper also discusses a clustering-based method for patient feature partitioning.

This paper is organized as follows. Section ‘Preliminary’ presents preliminary knowledge used in this paper. Section ‘Method’ describes the development of the genetic-fuzzy system for UA risk assessment. Experimental studies of the performance of the proposed approach are presented in Section ‘Results and discussion’. Section ‘Conclusion’ concludes the paper.

## Preliminary

Let *D* = {*σ*_1_,⋯,*σ*_*n*_} be a patient data-set consisting of a finite set of UA patient cases. Let *A* = {*a*_1_,⋯,*a*_*n*_} represent all patient features that appear in *D* and *Class* = {*low-risk*, *medium-risk*, *high-risk*} be a set of UA risk levels. Each feature *a* may have a categorical or numerical underlying domain, denoted *dom*(*a*). Each patient case *σ* (*σ* ∈ *D*) contains values of some patient features from *A*. Let *σ*(*a*) (*σ*(*a*) ∈ *dom*(*a*)) be the target feature value for the patient case *σ* for feature *a*.

For example, Table 1 shows an example patient data-set, which consists of five patient cases. Each case contains 1 numerical patient features (i.e., *age*) and 3 categorical patient features (i.e., *sex*, *smoking*, and *has event recently*).

**Table 1:** An example patient data-set

	**Age (year)**	**Sex**	**Smoking**	**Heart events recently**	**Physician assessment**
*σ*_1_	74	Male	No	Yes	Medium-risk
*σ*_2_	81	Female	No	Yes	Medium-risk
*σ*_3_	74	Male	Yes	Yes	High-risk
*σ*_4_	71	Male	No	Yes	High-risk
*σ*_5_	76	Female	No	No	Low-risk
*σ*_6_	67	Male	Yes	No	Medium-risk

The cases are simplified information extraction from patient records of Chinese PLA general hospital.

For numerical patient feature *a* (*a* ∈ *A*), let $\left\{l_{a}^{1},l_{a}^{2},\cdots ,l_{a}^{m}\right\}$ be a set of linguistic terms defined over *a*. Let *μ*_*a*,*j*_(*σ*(*a*)) be the membership degree on the value of a feature *a* of the patient case *σ* to the fuzzy set corresponding to the linguistic label $l_{a}^{j}$ for this feature *a*. Note that the degree of membership of each value of *a* in any of the fuzzy sets specified for *a* is directly based on the evaluation of the membership function of the particular fuzzy set with the value of *a* as input. The fuzzy partition of *dom*(*a*) is composed of $\left\{l_{a}^{1},\cdots ,l_{a}^{m}\right\}$ that satisfies $\sum_{j=1}^{m}\mu_{a,j}(x)=1,\forall x\in \text{dom}(a)$.

In this study, we employ fuzzy rules of the following form [9,12]:

(1) r:Cond→Class

where *Cond* is the antecedent part of the rule, and *Class* is the consequent part of the rule. For example, a fuzzy rule can be expressed as:

(2) $$R:\text{IF}\;a_{1}\;\text{is}\;(l_{a}^{1}\;\text{or}\;l_{a}^{3}\;)\;\text{and}\;a_{5}\;\text{is}\;l_{a}^{4}\;\text{THEN}\;\text{low-risk}.$$

It must be noted that any subset of the complete set of patient features, with any combination of linguistic labels related to the operators *and* and *or*, can take part in the rule antecedent. For this kind of fuzzy rule, we say that a patient case *σ* supports the antecedent part of a rule *r* if

(3) $$\begin{array}{l} \text{APC}(\sigma ,r)=\frac{1}{n}\overset{n}{\underset{i=1}{\sum }}\max\{\mu_{a_{i},1}(\sigma (a_{i})),\cdots ,\mu_{a_{i},m}(\sigma (a_{i}))\}>0 \end{array}$$

where $\mu_{a_{i},j}(\sigma (a_{i}))$ is the membership degree of patient feature *a*_*i*_ for *σ* to the fuzzy set corresponding to the linguistic label $l_{a_{i}}^{j}$ for *a*_*i*_; and *APC* is the antecedent part compatibility between a patient case and the antecedent part of a fuzzy rule. For the categorical features, the degrees of membership are zero or one.

For a patient case *σ*, the support degree of *σ* by a specific rule *r* is calculated as follows:

(4) $$\text{Supp}(\sigma ,r)=\left\{\begin{array}{cc} \text{APC}(\sigma ,r) & \text{the actual risk level of}\;\sigma \;\text{is equal to the class of}\;r\\ 0 & \text{otherwise} \end{array}\right)$$

In general, a fuzzy rule can be considered to be a classification rule if the antecedent contains fuzzy item sets, and the consequent part contains only one class label such as *low-risk, medium-risk*, or *high-risk* in this study. A fuzzy rule *r* : *Cond* → *Class* could be measured directly in terms of support and confidence as follows:

(5) $$\text{Support}(r)=\frac{\sum_{\sigma \in D}\text{Supp}(\sigma ,r)}{\left|D\right|}$$

(6) $$\text{Confidence}(r)=\frac{\sum_{\sigma \in D}\text{Supp}(\sigma ,r)}{\sum_{\sigma \in D}\text{APC}(\sigma ,r)}$$

## Method

**Figure 1:**
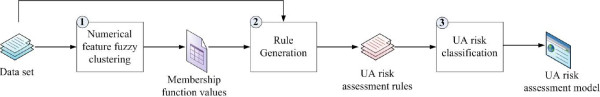
The main steps of the proposed UA risk assessment model.

In this section, we describe the process of utilizing GFS to develop an intelligent system for the problem of UA risk assessment. As shown in Figure 1, the proposed method consists of three steps. At first, all the numerical patient features of the data-set are given as input for the fuzzy clustering module for calculating the membership functions. Then, calculated function values are given to the rule generation module for obtaining UA risk assessment rules. Based on the derived rules, a classification model for UA risk assessment is generated.

The case study was performed in the Cardiology Department at the Chinese PLA General hospital. Prior approval was obtained from the data protection committee of the hospital to conduct the study. We state that the patient data was anonymized in this study and in the Method section of this paper.

### Fuzzy clustering for numerical feature discretization

One of the most important steps in UA risk assessment is to deal with the intrinsic uncertainty about the variables utilized. As described in [13], fuzzy set is a common tool for facilitating the interpretation of rules in linguistic terms, and avoiding unnatural boundaries in the partitioning of the variable domains. It is especially useful in clinical settings where the boundaries of a piece of information used may not be clearly defined. Regarding our task of UA risk assessment, the quality of the results produced relies quite crucially on the appropriateness of fuzzy sets to the given patient features. So, fuzzy sets must be consistent with the values of the corresponding feature.

Fuzzy sets can be provided by physicians. However, the provided fuzzy sets by physicians may not be suitable for mining fuzzy association rules from data-set. Also, it is extremely difficult for physicians to estimate the most appropriate fuzzy sets. In order to cope with these problems, we first concentrate on how fuzzy sets of the given features are determined automatically from the collected data-set. Clustering techniques are usually employed as a preprocessing step to partition numerical features [14]. In this study, we employed a hierarchical agglomerative clustering [15-17] algorithm to partition numerical features.

As shown in algorithm 1, hierarchical agglomerative clustering begins with each value as a separate cluster and merges them into successively larger clusters. The process is repeated until the similarity between any pair of clusters is less than a threshold value *ε*. Consequently, the algorithm builds a structure called dendogram, i.e., a tree illustrating the merging process and intermediate clusters. Similarity between two clusters *c*_1_ and *c*_2_ can be measured as follows:

(7) $$\text{sim}(c_{1},c_{2})=\frac{1}{\left|c_{1}||c_{2}\right|}\underset{x\in c_{1}}{\sum }\underset{y\in c_{2}}{\sum }|x-y|$$

where |*c*_1_| and |*c*_2_| are the number of clusters *c*_1_ and *c*_2_, respectively.

**Algorithm 1** Hierarchical Agglomerative Clustering

This way, the values of each patient feature in the data-set are distributed over a set of derived clusters using Algorithm 1. For each patient feature, the centroids of the clusters are the set of midpoints of the fuzzy sets. To illustrate the process, suppose we want to find fuzzy sets for a specific patient feature *a*, which is quantitative with a range from min(*dom*(*a*)) to max(*dom*(*a*)). Let $\left\{v_{a}^{1},\cdots ,v_{a}^{m}\right\}$ be the set of mid-points of the fuzzy sets for *a*. As a result, the derived fuzzy sets will have the following ranges: $\left[v_{a}^{0},v_{a}^{1}],[v_{a}^{0},v_{a}^{2}],\cdots ,[v_{a}^{m-1},v_{a}^{m+1}\right]$ and $\left[v_{a}^{m},v_{a}^{m+1}\right]$, where $v_{a}^{0}=\min(\text{dom}(a))$, and $v_{a}^{m+1}=\max(\text{dom}(a))$.

After the fuzzy sets of each numerical feature are obtained, the corresponding membership function can be generated for each fuzzy set. In this study, we used membership functions of both semi-trapezoidal shape and triangular shape because they are in general the most appropriate shapes and the most widely used in fuzzy systems. For example, for the fuzzy set with a range from $v_{a}^{0}$ to $v_{a}^{1}$, the membership function is given by

(8) $$\mu_{a,0}(x)=\left\{\begin{array}{cc} 1 & \text{if}\;x<v_{a}^{0}\\ \frac{v_{a}^{1}-x}{v_{a}^{1}-v_{a}^{0}} & \text{if}\;v_{a}^{0}\leq x\leq v_{a}^{1}\\ 0 & \text{if}\;x>v_{a}^{1} \end{array}\right)$$

For each fuzzy set with midpoint $v_{a}^{j}$, where 1 ≤ *j* ≤ *m*, the membership function is given by

(9) $$\mu_{a,j}(x)=\left\{\begin{array}{cc} \frac{x-v_{a}^{j-1}}{v_{a}^{j}-v_{a}^{j-1}} & \text{if}\;v_{a}^{j-1}\leq x\leq v_{a}^{j}\\ \frac{v_{a}^{j+1}-x}{v_{a}^{j+1}-v_{a}^{j}} & \text{if}\;v_{a}^{j}\leq x\leq v_{a}^{j+1}\\ 0 & \text{otherwise} \end{array}\right)$$

And for the fuzzy set with a range from $v_{a}^{m}$ to $v_{a}^{m+1}$, the membership function is given by

(10) $$\mu_{a,m+1}(x)=\left\{\begin{array}{cc} 1 & \text{if}\;x>v_{a}^{m+1}\\ \frac{x-v_{a}^{m}}{v_{a}^{m+1}-v_{a}^{m}} & \text{if}\;v_{a}^{m}<x\leq v_{a}^{m+1}\\ 0 & \text{if}\;x\leq v_{a}^{m} \end{array}\right)$$

**Figure 2:**
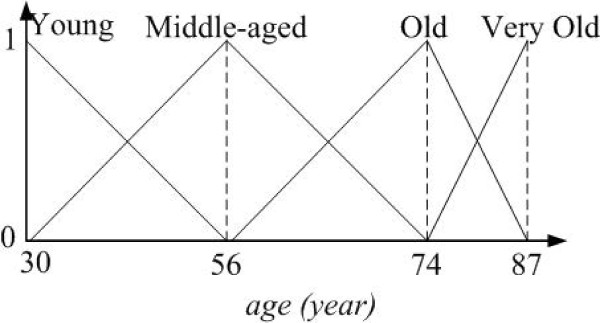
**The membership functions for patient feature,** ***age*.**

For example, given a numerical feature, say *age* with three different ranges, i.e., [30, 56], [56, 74], and [74, 87]. The values of *age* range from 30 to 87, and can be classified into four fuzzy sets, as shown in Figure 2.

### Fuzzy association rule mining

This subsection presents a GFS for mining fuzzy association rules from a data-set. The proposed system uses fuzzy rule format defined in Equation (2), which offers a flexible structure to the rules, allowing each patient feature to take more than one value and facilitating the extraction of general UA risk assessment rules.

#### Chromosome representation

As mentioned above, a US risk assessment rule *r* consists of an antecedent part *Cond* and a consequent part *Class*. In this study, we code the antecedent part *Cond* of *r* as one chromosome consisting of a set of segments. Each segment corresponds to a specific patient feature. The set of possible values for the categorical features is that indicated by the problem, and for numerical features, it is the set of linguistic terms determined by the clustering method presented above. The consequent part *Class* of *r* is prefixed to one of the possible values of risk levels, i.e., *high-risk*, *medium-risk*, and *low-risk*.

Table 2 describes a representation for a rule with numerical and categorical features for the values of a specific risk level *high-risk*. Note that a bit for each one of the possible values of each feature is stored. In this way, if the value of the corresponding element is 0, it indicates that the value is not used in the rule. Otherwise, if the value is 1, it indicates that the corresponding value is included. If a rule contains all the elements corresponding to a feature of the value 1, or all of them contain the value 0, this indicates that this feature has no relevance for the information contributed in the rule, and so this feature is ignored. In these cases, the feature does not take part in the rule. For example, as shown in Table 2, the rule is represented by a binary string 〈(0011)(00)(10)(10) : *high-risk*〉, where parentheses are to separate segments, and ":" is to separate the IF part and the THEN part of the rule. In this example, *a*_1_ has four possible values and *a*_2_, *a*_3_, and *a*_4_ have two possible values. Note that *a*_2_ does not take part in the rule as *a*_2_ takes none of its values, and thus *a*_2_ is irrelevant for the rule. This binary string can be interpreted as the following rule: "IF *age* is (*old* or *very old*) and *smoking* is *true* and *has heart events recently* is *true* THEN UA risk is *high*".

**Table 2:** Representation of a fuzzy rule with numerical and categorical features in UA risk assessment

**Patient feature**	**Age**				**Sex**		**Smoking**		**Has heart events recently**		
**Linguistic label**	Young	Middle-aged	Old	Very old	Male	Female	Yes	No	Yes	No	
**Rule**	0	0	1	1	0	0	1	0	1	0	High-risk

#### Fitness function and selection process

The objective of this step is to find the accurate and general rules for UA risk assessment. Thus, given a specific rule *r*, the GA method uses the composite fitness function consisting of support and confidence in the following way:

(11) $$\text{Fitness}(r)=\frac{\text{Support}(r)+\text{Confidence}(r)}{2}$$

The objective of the fitness function is defined as the composite measure of support and confidence. This composite measurement provides an effective selection environment which balances the accuracy and generality of the rules.

Three operators, i.e., selection, crossover, and mutation, are applied in the proposed GA method to generate the offspring population, which are illustrated as follows: 

• The selection procedure is used for evolution where two individuals are selected randomly from the current population and used for crossover and mutation operators. During each generation, individuals with higher fitness values survive while those with lower fitness values are destroyed.

• Two parents are selected and recombined according to the predefined crossover probability during crossover. In this work, one point crossover is applied due to its simplicity, which can randomly select different cutoff points for each parent to generate offspring rule sets.

• Each element in the chromosome is applied to mutation with a predefined mutation probability. The value of a randomly selected element is converted to 0 if its value is 1, and vice versa. Elimination of existing rules and addition of new rules can also be used as mutation operations. As a result, the number of rules in the rule sets string can be changed accordingly.

During GA operations, redundant rules might be produced. For example, we say a rule "If Age is (Young) and ST is (Low) then Risk is (Low)" is redundant w.r.t the other rule "If Age is (Young OR Meddle-ages) and ST is (Low) then Risk is (Low)", if both rules have the same support degree on a data-set. Thus, the proposed algorithm must check the rule sets and maintains single among all the rules, to guarantee the consistency of fuzzy systems. The stopping criterion of the proposed algorithm is the number of generations. The scheme of the proposed algorithm is shown in Algorithm 2.

**Algorithm 2** The GA-based rule mining algorithm for UA risk assessment.

Taking the data set shown in Table 1 as an example, and assuming the population size as 50, the number of generation as 1000, the crossover rate as 0.5, and the mutation rate as 0.2 in the proposed genetic algorithm, we can obtain an example rule-set, as shown in Table 3.

**Table 3:** **Rules derived from the example patient data-set shown in Table**1

**# Rule**	**Rule**	**Support**	**Confidence**	**Fitness**
1	IF Age (Young) AND Heart Events Recently is (False) THEN Risk is Low	0.083	0.5	0.291
2	IF Age (Young) AND Sex is (Female) AND Smoke is (False) THEN Risk is Low	0.11	0.33	0.22
3	IF Age is (Young OR Meddle-aged) AND Smoke is (False) AND Heart Events Recently is (True) THEN Risk is Medium	0.244	0.514	0.378
4	IF Age is (Old) AND Smoke is (False) THEN Risk is Medium	0.339	0.465	0.402
5	IF Age is (Old OR Very-Old) AND Smoke is (True) THEN Risk is High	0.269	0.377	0.323
6	IF Age is (Meddle-aged OR Old OR Very-Old) AND Sex is (Male) AND Heart Events Recently is (True) THEN Risk is High	0.324	0.455	0.390

### UA risk assessment model

Based on the derived rule set, we can generate a classification model for UA risk assessment. Formally, let ℜ be the set of derived UA risk assessment rules. For each *r* : *Cond* → *Class* (*r* ∈ ℜ), the score value of the target *class* (*class* ∈ {*low-risk*,*medium-risk*,*high-risk*}) of *r* with respect to a given patient case *σ* can be assessed, by using the following equation:

(12) $$v_{\text{class}}=\underset{r\in ℜ}{\sum }\text{Confidence}(r)\beta (r,\sigma )$$

where *Confidence*(*r*) is the confidence value of rule *r*, and *β*(*r*,*σ*) is the firing strength of the input patient case *σ* on the antecedent part of rule *r*.

The firing strength *β*(*r*,*σ*) is defined as

(13) $$\begin{array}{l} \beta (r,\sigma )=\;\underset{\langle (a,l_{a}^{1}),\cdots ,(a,l_{a}^{m})\rangle \;\in \;\text{Cond}}{\sum }\;\max\{\mu_{a,1}(\sigma (a)),\cdots ,\mu_{a,m}(\sigma (a_{i}))\} \end{array}$$

where *μ*_*a*,*j*_ represents the fuzzy membership function for the pair of *a* and the linguistic term $l_{a}^{j}$ in the $\langle (a,l_{a}^{1}),\cdots ,(a,l_{a}^{m})\rangle (1\leq j\leq m)$ in the antecedent part *Cond* of rule *r*.

With respect to the target values of risk levels, i.e., *low-risk, medium-risk, high-risk*, the corresponding scores *v*_*l*_,*v*_*m*_,*v*_*h*_ can be generated based on Equation (12). And the risk level with the top score in the scoring vector *v* will be the predicted risk level for the patient case *σ*.

(14) $${\hat{\text{Class}}}_{\sigma }=\text{argmax}(\{v_{l},v_{m},v_{h}\})$$

Taking *σ*_1_ shown in Table 1 as an example, and using the derived rule-set shown in Table 3, the score values for *σ*_1_ are calculated as *v*_*l*_ = 0.4, *v*_*m*_ = 2.088, and *v*_*h*_ = 1.745, by Equation (12). Thus, the predicted risk level for *σ*_1_ is *medium-risk*.

## Results and discussion

To evaluate the feasibility of the presented methods, a clinical case study is conducted through the cooperation with the Cardiology Department of the Chinese PLA General Hospital. The data-set collected from the hospital consists of 54 patient cases. The target classes of UA risk levels include: *low-risk*, *medium-risk*, and *high-risk*. Physicians that evaluated these cases are experienced clinicians working for the hospital with 10 years of working experience on average. As a result, 16 cases are classified into the *low-risk* group, 33 cases are classified into the *medium-risk* group, and 5 cases are classified into the *high-risk* group, respectively. Patient features (9 numerical features and 17 categorical features) that appear in the data-set are shown in Table 4. These features are regularly recorded in UA treatment practice.

**Table 4:** Patient features utilized in UA risk assessment

**Numerical feature**	
**Name**	**Fuzzy sets**
Age	{Young, middle-aged, old, very old}
Systolic Blood Pressure (SBP)	{Low, medium, high}
Diastolic Blood Pressure (DBP)	{Low, low-medium, high-medium, high}
Creatinine	{Low, medium, high}
AST	{Low, medium, high}
LDH	{Low, medium, high}
CK	{Low, medium, high}
CK MB	{Very low, low, medium, high}
CnT	{Low, high}
**Categorical feature**	
**Name**	**Crisp sets**
Sex	{Male, female}
Smoke	{Yes, no}
Atrial premature beat	{Yes, no}
Significant change in ST in ECG	{Yes, no}
Has heart events recently	{Yes, no}
Anamnesis of coronary heart disease	{Yes, no}
Anamnesis of renal insufficiency	{Yes, no}
Anamnesis of bleeding	{Yes, no}
Anamnesis of diabetes	{None, type 1, type 2}
Anamnesis of hyperlipaemia	{Yes, no}
Anamnesis of hypertension	{None, level I, level II, level III}
Has aspirin in recent 7 days	{Yes, no}
Has percutaneous coronary intervention	{Yes, no}
Lungs event as expectoration with blood	{Yes, no}
Change in T	{Yes, No}
Heart event by drinking	{Yes, no}
Irregular pulse	{Yes, no}

All experiments were performed on a Lenevo Compatible PC with an Intel Pentium IV CPU 2.8 GHz, 4G byte main memory running on Microsoft Windows 7. The algorithms were implemented using Microsoft C#. A 10-fold cross-validation was performed to evaluate the proposed method, by using a 90% of the data-set as the training set, and the remaining 10% as the validation set. To reduce variability, 10 rounds of this validation process were performed by using different partitions.

**Figure 3:**
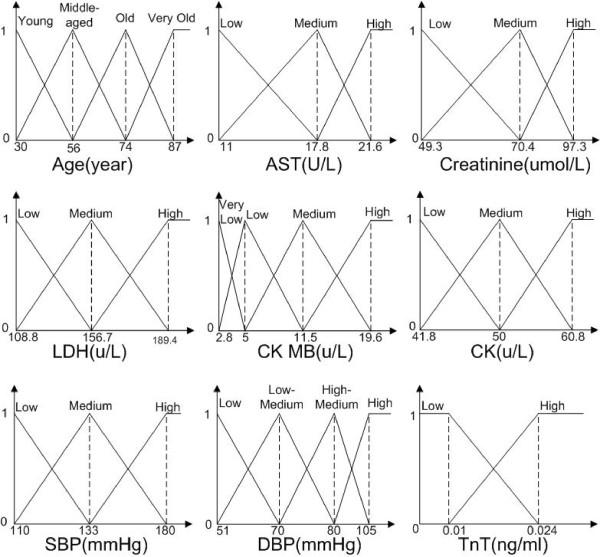
Membership function of input numerical patient features.

For the first step of patient feature discretization, we applied Hierarchical Agglomerative Clustering method to each numerical feature to generate a set of fuzzy sets. The derived fuzzy sets of input numerical patient features are shown in Figure 3.

To mine fuzzy association rules, we have taken the population size as 100, the number of generation as 1000, the crossover rate as 1.0, and the mutation rate as 0.2 in the proposed genetic algorithm. By using these parameters, we run our genetic algorithm for each target class of UA risk level, one by one, to obtain a set of fuzzy rules for a given class.

Table 5 shows the rules obtained, which have the best fitness values for the target classes of UA risk level (i.e., *low-risk*, *medium-risk*, and *high-risk*). In this table, the number of patient features involved in each rule (# of Feature.), and the Support and Confidence of each rule are shown. The values of Support and Confidence are between zero and one. High values in support means that the rule covers most of patient cases which are categorized into the class, and high values in confidence means that the rule has few negative patient cases [12]. Note that the knowledge discovered for each target value of risk level is understandable by physicians due to the use of fuzzy logic and the low number of rules and conditions in the rule antecedents (below 40% of 26 patient features). Tables 6, 7 and 8 show the rules obtained which have the best fitness values corresponding to the target classes of risk level.

**Table 5:** **Results for *low-risk, medium-risk *, and** ***high-risk***

**Risk level**	**# of feature**	**Support**	**Confidence**
Low	9	0.204	0.375
	8	0.195	0.380
Medium	10	0.448	0.633
	10	0.449	0.631
	10	0.067	0.114
High	9	0.064	0.115

**Table 6:** **Rules for** ***low-risk***

**# Rule**	**Rule**
1	IF Age is (Young OR Meddle-aged) AND AST is (Low OR Medium) AND Creatinine is (Low OR Medium) AND CK is (Low OR Medium) AND LDH is (Low OR Medium) AND CK MB is (Medium) AND has PCI is (No) AND Hypertension is (No OR Level I OR Level II) AND Arrhythmia is (No) THEN Risk is Low
2	IF Age is (Young OR Meddle-aged) AND AST is (Low OR Medium) AND Creatinine is (Low OR Medium) AND CK is (Low OR Medium) AND LDH is (Low OR Medium) AND CK MB is (Medium) AND has PCI is (No) AND Hypertension is (No OR Level I OR Level II) THEN Risk is Low

**Table 7:** **Rules for** ***medium-risk***

**# Rule**	**Rule**
1	IF Age is (Old) AND Creatinine is (Low OR Medium) AND CK is (Low OR Medium) AND LDH is (Medium) AND SBP is (Medium OR High) AND Heart Events Recently is (Yes) AND Aspirin in Recently 7 days is (No) AND has PCI is (Yes) AND Hypertension is (Level I OR Level II OR Level III) AND Hyperlipaemia is (No) THEN Risk is Medium
2	IF Age is (Middle-aged OR Old) AND Creatinine is (Low OR High) AND CK is (Low OR High) AND LDH is (Medium) AND SBP is (Medium OR High) AND Heart Events Recently is (Yes) AND Aspirin in Recently 7 days is (No) AND has PCI is (Yes) AND Hypertension is (Level I OR Level II OR Level III) AND Hyperlipaemia is (No) THEN Risk is Medium

**Table 8:** **Rules for** ***high-risk***

**# Rule**	**Rule**
1	IF Age is (Old OR Very-Old) AND AST is (High) AND Creatinine is (High) AND CK is (Medium) AND LDH is (Medium OR High) AND CK MB is (High) AND SBP is (Low OR Medium) AND Smoke is (Yes) AND Hypertension is (Level II OR Level III) AND Diabetes is (Type 1 OR Type 2) THEN Risk is High
2	IF Age is (Old OR Very-Old) AND AST is (High) AND Creatinine is (High) AND CK is (Medium) AND LDH is (Medium OR High) AND CK MB is (High) AND SBP is (Low OR Medium) AND Hypertension is (Level II OR Level III) AND Diabetes is (Type 1 OR Type 2) THEN Risk is High

Now, acquiring fuzzy rule base, it is possible to complete UA risk assessment through the proposed classification model. As we mentioned above, the ensemble of fuzzy rules and the proposed classification model perform the role of a mathematical function to obtain the system output. This output is the stratification of UA risk. This way, for each patient whose data are informed as system inputs, the most likely risk level for that patient is generated.

**Figure 4:**
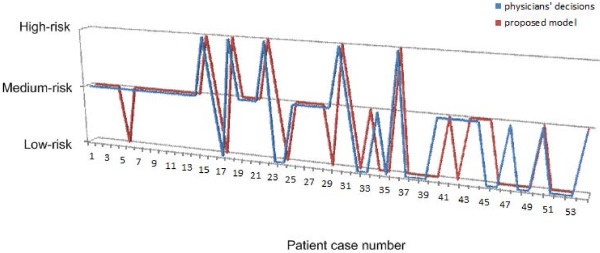
Comparison between the proposed model and physicians’ decisions.

The comparison between the proposed model and physicians’ decisions is done for each partition of data-set. The comparison is given in Figure 4. The proposed system made the same decisions as the physician in 46 (out of a total of 54) tested cases (85.2%). From Figure 4, the proposed model did not predict the risk well in the case of the fifth (the physician assessment is medium-risk while the proposed model assessment is low-risk), the twenty third (the physician assessment is low-risk while the proposed model assessment is medium-risk), the twenty ninth (the physician assessment is medium-risk while the proposed model assessment is low-risk), the thirty third (the physician assessment is low-risk while the proposed model assessment is medium-risk), the thirty fourth (the physician assessment is medium-risk while the proposed model assessment is low-risk), the forty (the physician assessment is medium-risk while the proposed model assessment is low-risk), the forty second (the physician assessment is medium-risk while the proposed model assessment is low-risk), and the forty seventh (the physician assessment is medium-risk while the proposed model assessment is low-risk).

Furthermore, we measure the accuracy of the proposed approach using the sum of two performance measures: sensitivity (probability that the test correctly classifies a case with a specific risk level) and specificity (probability of correctly classifying a case without a specific risk level).

(15) $$\text{Sensitivity}(v_{\text{class}})=\frac{\sum_{i=1}^{n}\text{TP}(\sigma_{i})}{\sum_{i=1}^{n}\text{TP}(\sigma_{i})+\sum_{i=1}^{n}\text{FN}(\sigma_{i})}$$

(16) $$\text{Specificity}(v_{\text{class}})=\frac{\sum_{i=1}^{n}\text{TN}(\sigma_{i})}{\sum_{i=1}^{n}\text{TN}(\sigma_{i})+\sum_{i=1}^{n}\text{FP}(\sigma_{i})}$$

where *v*_*class*_ ∈ {*low-risk*, *medium-risk*, *high-risk*}; *TP* is the set True Positive, patient cases with the specific risk level *v*_*class*_ classified correctly; *FN* is the set False Negative, patient cases with the specific risk level *v*_*class*_ classified as other risk levels; *TN* is the set True Negative, patient cases without the specific risk level *v*_*class*_ classified; *FP* is the set False Positive, patient cases without the specific risk level *v*_*class*_ classified as *v*_*class*_. In Table 9 the sensitivity and specificity obtained for each risk level are presented. The experimental results indicate that the proposed method is feasible for predicting risk levels of unstable angina patients.

**Table 9:** Sensitivity and specificity with different risk levels

	**Sensitivity**	**Specificity**
Low-risk	0.8125	0.8421
Medium-risk	0.8182	0.85
High-risk	1.0	1.0

## Conclusion

In this paper, we have presented an intelligent system for UA risk assessment by combining genetic algorithm and fuzzy association rule mining. The developed approach has been tested on a data-set consisting of 54 UA patient cases from the Cardiology department of Chinese PLA General hospital. The experimental results show that considerable agreement is achieved between the proposed approach and physicians’ problem solving knowledge.

The main novelty of the developed model is that it represents a valuable objective tool for UA risk assessment. In medical literature, physicians are in discrepancies about the risk factors highlighted. This research has focused on the application of computational intelligence. In particular, a genetic-fuzzy system, to identify the key factors behind UA, is proposed, which could be used for educational purposes, and with further improvements, could assist and guide young physicians in their daily work.

For future studies, there may be a comparison of effectiveness in terms of the proposed system with traditional UA risk assessment models, such as TIMI, GRACE, etc. The application of the proposed system to other kinds of CVD, such as heart failure, will also be investigated. Furthermore, other computational intelligence techniques can be associated with the developed system.

## Competing interests

The authors declare that they have no competing interests.

## Authors’ contributions

WD: conception, design, analysis of data, development, interpretation and evaluation of results, and drafting of the manuscript. ZH: conception, design, and development of methods, drafting of the manuscript, and critical revision of manuscript. LJ: conception, design, extraction of data, interpretation and evaluation of results. HD: design of methods, analysis of data, and critical revision of manuscript. All authors read and approved the final manuscript.

## Pre-publication history

The pre-publication history for this paper can be accessed here:

http://www.biomedcentral.com/1472-6947/14/12/prepub
